# Characterization of the Immune Response of Atlantic Salmon to *Tenacibaculum dicentrarchi* Using In Vivo and In Vitro Approaches

**DOI:** 10.3390/ani16182908

**Published:** 2026-09-16

**Authors:** Débora Torrealba, Cristobal Domínguez, Paula Valenzuela-Avilés, José Gallardo-Matus, Luis Mercado, Marcos Mancilla

**Affiliations:** 1 Laborarorio de Nanobiotecnología e Inmunología Marina, Instituto de Ciencias de la Ingeniería, Universidad de O’Higgins, Avenida Libertador Bernardo O’Higgins 611, Rancagua 2841959, Chile; 2 Programa de Doctorado en Biotecnología, Pontificia Universidad Católica de Valparaíso, Valparaíso 2373223, Chile; cdoming@espol.edu.ec; 3 Programa de Doctorado en Biotecnología, Universidad Técnica Federico Santa María, Valparaíso 2373223, Chile; 4 Centro Nacional de Acuicultura e Investigaciones Marinas, CENAIM, Escuela Superior Politécnica del Litoral, ESPOL, Polytechnic University, Campus Gustavo Galindo Km. 30.5 Vía Perimetral, Guayaquil P.O. Box 09-01-5863, Ecuador; 5 Laboratorio de Genética y Genómica Aplicada, Escuela de Ciencias del Mar, Pontificia Universidad Católica de Valparaíso, Avenida Universidad 330, Valparaíso 2373223, Chile; paula.valenzuela.a@mail.pucv.cl (P.V.-A.); jose.gallardo@pucv.cl (J.G.-M.); 6 Grupo de Marcadores Inmunológicos en Organismos Acuáticos, Instituto de Biología, Pontificia Universidad Católica de Valparaíso, Avenida Universidad 330, Valparaíso 2373223, Chile; luis.mercado@pucv.cl; 7 ADL Diagnostic Chile, Puerto Montt 5480000, Chile; mmancilla@adldiagnostic.cl

**Keywords:** tenacibaculosis, immersion challenge, bath challenge, head kidney macrophages, salmonids

## Abstract

Tenacibaculosis, primarily caused by *Tenacibaculum dicentrarchi* in Chile, is now the second leading infectious cause of mortality in Chilean Atlantic salmon (*Salmo salar*) farming, yet the host immune response remains uncharacterized. Here, we combined in vitro and in vivo approaches to characterize the immunopathological interaction between Atlantic salmon and a Chilean *T. dicentrarchi* isolate. Primary head kidney macrophages stimulated with live bacteria showed early and sustained upregulation of *tnfα*, *il1β*, and *il8* (4–24 h), followed by late induction of *inos* at 24 h, consistent with a classical M1-like proinflammatory profile. In parallel, *il10* was robustly upregulated, while M2 marker *arg* was downregulated, reflecting balanced immunoregulation with active suppression of alternative activation. Production of TNF-α and IL-10 proteins was confirmed by immunofluorescence. An immersion challenge validated pathogenic relevance, yielding 32.4% cumulative mortality, with peak deaths at day 4 and a clinical course matching field outbreaks, including frayed fins, skin ulcers, mouth damage, and hemorrhage, with partial lesion resolution by day 28. Together, these results provide the first integrated in vitro–in vivo characterization of the Atlantic salmon response to *T. dicentrarchi*, combining cellular immune response profiling with experimental disease progression, and establishing a reproducible approach that captures both acute and recovery phases of disease, supporting future vaccine and therapeutic development against tenacibaculosis.

## Simple Summary

Salmon farming is one of Chile’s most important food industries, but bacterial diseases threaten its productivity and sustainability. One of the most damaging is tenacibaculosis, caused in Chile primarily by *Tenacibaculum dicentrarchi*, a bacterium that produces severe skin lesions in Atlantic salmon and is currently the second leading infectious cause of death on Chilean marine farms. Despite its growing impact, how salmon defend themselves against this pathogen was completely unknown. In this study, we used two complementary approaches to fill this gap: we exposed immune cells isolated from salmon kidneys to live bacteria in the laboratory, and we infected whole fish by immersing them in a bacterial solution to mimic natural conditions. The laboratory results show that salmon immune cells respond rapidly and strongly, producing both molecules that attack the bacteria and molecules that limit tissue damage. The fish challenge confirmed that infection caused disease signs identical to those seen on farms, with about one-third of fish dying, mostly within the first week; however, surviving fish progressively recovered over the following weeks. Together, these findings provide the first description of how Atlantic salmon fight *T. dicentrarchi*, establishing an experimental approach that can be used to develop vaccines and treatments to protect salmon against tenacibaculosis.

## 1. Introduction

*Tenacibaculum dicentrarchi* is a broad-host-range pathogen of marine fish, first described from European sea bass (*Dicentrarchus labrax*) in 2012 [1]. Since then, it has been reported in multiple species, including Atlantic salmon (*Salmo salar*) [2], Atlantic cod (*Gadus morhua*) [3], conger eel (*Genypterus chilensis*) [4], and palm ruff (*Seriola violacea*) [5], with cases documented across Europe, South America, and Oceania. This pathogen is a Gram-negative bacterium [6] with a long, thin, rod-shaped morphology and the ability to glide and form biofilms [7]. Infection causes an ulcerative skin disorder characterized by skin ulcers that frequently expose underlying musculature, mouth rot presenting as tissue erosion, gill ulceration or hemorrhage, and, in some cases, lesions with a distinctive yellow margin and fraying of fins and tails, ultimately resulting in significant mortality [2,8,9].

In Chile, tenacibaculosis has emerged as a significant disease in marine salmon farming, with a notable and sustained increase in associated mortalities since 2017 [10]. Since then, this pathogen has become the second-leading infectious cause of mortality in marine farms, after *Piscirickettsia salmonis* [11]. While *Tenacibaculum maritimum* and *Tenacibaculum finnmarkense* have also been identified as affecting salmonid populations [2,12,13,14], *T. dicentrarchi* is considered the primary cause of tenacibaculosis-related mortalities in Chile [15].

Salmon are not equally susceptible to tenacibaculosis [2], and while exposure may lead to infection across species, the resulting clinical disease severity depends on a combination of environmental conditions, host health status, and bacterial strains [8,16,17]. Several predisposing factors recur consistently across the literature. Sea transfer and small post-smolt size represent a critical vulnerability window, as many outbreaks occur shortly after entry into seawater at a stage when susceptibility to *Tenacibaculum* infection is particularly high [9,16,18]. Low water temperatures, especially during late winter and spring, are repeatedly associated with tenacibaculosis outbreaks in Atlantic salmon, particularly in the period immediately following sea transfer [16,19]. Physical damage to the skin or mucus layer, caused by handling, crowding, or net contact, further facilitates infection, as ulcers typically originate at sites of prior injury and experimental challenges often require epidermal disruption to establish disease [9]. The integrity of the mucus barrier and skin microbiome also plays a central role: *T. dicentrarchi* adheres to and proliferates within skin mucus, and infection alters both the skin and gut microbiota, reflecting compromised barrier and immune function [20,21,22]. Stress associated with capture, handling, and crowding contributes further, with affected fish exhibiting lethargy, loss of balance, and rapid ulcer progression [9]. Finally, virulence differences among bacterial strains must be considered, as *T. dicentrarchi* isolates vary considerably in pathogenicity, with some isolates causing high mortality under bath-challenge conditions, while others produce only mild disease [2,23].

The immune response of fish to tenacibaculosis has been characterized across several host–pathogen models, predominantly involving *T. maritimum* in species such as Atlantic salmon, European sea bass, turbot, Senegalese sole, and batfish [20,24,25,26]. The first line of defense relies on the mucosal barrier [27], where skin mucus components, including lysozyme, proteases, antiproteases, and complement, provide initial bactericidal activity; however, *Tenacibaculum* can adhere to and proliferate within this medium, overcoming early defenses in Atlantic salmon [20]. Locally, infection rapidly triggers inflammatory responses, with upregulation of pro-inflammatory genes (*il1β*, *il6*, *il8*, *tnfα*) in skin, gills, and intestine, accompanied by necrosis and intense leukocyte infiltration at lesion sites in European sea bass infected with *T. maritimum* [24,25]. In Senegalese sole, mucosal enzymatic defenses are initially suppressed within the first 48 h post-challenge, recovering and increasing significantly only by 14 days post-challenge [28]. At the systemic level, infected fish commonly develop neutrophilia, monocytosis, and altered plasma bactericidal and lysozyme activities, while the head kidney upregulates both pro-inflammatory cytokines (*il1β*, *il6*, *il8*) and regulatory mediators such as *tgf-β*, indicating simultaneous inflammatory and tissue repair responses [24,25,26]. Adaptive immune activation has also been documented, with *Tenacibaculum* antigens inducing specific antibody responses and enhanced phagocytosis in sea bass and turbot [29,30]. Furthermore, outer membrane vesicles (OMVs) produced by *T. maritimum* and *T. dicentrarchi* exhibit cytotoxic activity against head kidney macrophages, suggesting a mechanism for phagocyte evasion [30,31]. Despite these advances, the immune response of Atlantic salmon specifically to *T. dicentrarchi* remains uncharacterized.

The present study addresses this gap by combining in vitro and in vivo approaches to characterize the immunological and pathological interaction between Atlantic salmon and *T. dicentrarchi*. Specifically, we (i) determined the optimal stimulation conditions of Atlantic salmon head kidney macrophages with live *T. dicentrarchi*; (ii) analyzed the temporal modulation of M1- and M2-associated immune genes and validated cytokine expression at the protein level; and (iii) evaluated the temporal progression of clinical signs using an immersion challenge model. To our knowledge, this is the first study to characterize the macrophage response to *T. dicentrarchi* using a cellular approach, including primary head kidney macrophages and the SHK-1 cell line, while the in vivo component follows an immersion challenge approach consistent with that reported by Avendaño-Herrera.

## 2. Materials and Methods

### 2.1. Ethics Statement

All experimental procedures were conducted in accordance with the Chilean animal welfare regulations (Law No. 20,380. Animal Protection Law. Ministry of Justice: Santiago, Chile, 2009) and followed the ethical principles for animal experimentation established by the Comisión Nacional de Investigación Científica y Tecnológica (ANID). The study protocol was approved by the Bioethics Committees of the Universidad de O’Higgins and Pontificia Universidad Católica de Valparaíso (approval codes: CICUA 015-2024; BIOPUCV-BA 723-2024).

### 2.2. Source of Fish and Husbandry

Smoltified Atlantic salmon (*Salmo salar*) were provided by a hatchery in Puerto Montt (Los Lagos Region, Chile) for the in vitro study, and by Salmones Camanchaca’s Polcura hatchery (Biobío Region, Chile) for the in vivo study. A total of 208 fish were used in this study, comprising 15 fish (210.2 ± 26.4 gr bodyweight) for the in vitro experiments and 193 fish (185.4 ± 37.3 g bodyweight) for the in vivo procedures. Salmon from both hatcheries were transported in seawater using a custom-built transport vehicle with oxygen control and real-time monitoring to the Center for Sustainable Aquaculture of the Pontificia Universidad Católica de Valparaíso (Curauma, Valparaíso Region, Chile) for experiments. Health status was verified by qPCR screening for viral (ISAV and IPNV) and bacterial pathogens (*Vibrio ordalii*, *Flavobacterium psychrophilum*, *Tenacibaculum* spp., *P. salmonis*, and *Renibacterium salmoninarum*). Smoltified fish were distributed among four tanks of 500 L and held in recirculating aquaculture systems (RAS) containing seawater at 32 ppt salinity. They were maintained at an average temperature of 13.6 °C, with continuous monitoring of oxygen, water temperature, nitrogenous compounds, and pH. Fish underwent a 20-day acclimation period before experimental procedures, during which they were fed daily at 1% of their bodyweight with a commercial diet (44% protein and 21% lipid). Fish were not fed 24 h before manipulation. Euthanasia was performed at the end of the experiments or when required by anesthetic overdose (AQUI-S, 50 mL/100 L).

### 2.3. Source of T. dicentrarchi and Culture Conditions

An isolate of *T. dicentrarchi* (PM-90951) was obtained in 2018 from a water sample collected at a salmonid culture center in the Región de Los Lagos, southern Chile, and provided by ADL Diagnostic Chile (Puerto Montt, Chile). The isolate was cultured in marine agar and confirmed to be *T. dicentrarchi* by species-specific PCR (Table 1) and stored at −80 °C. The isolate was reactivated by streaking on Petri dishes containing *Flexibacter maritimus* medium (FMM) agar and incubated at 19 °C. After 48–72 h of incubation, colonies were transferred to 250 mL flasks containing 200 mL of FMM broth and cultured under constant shaking (110 rpm) for 24 h at 19 °C. Subsequently, 100 µL of the bacterial culture was transferred to another flask containing 100 mL of fresh FMM broth and incubated at 19 °C for 12 h with constant shaking. The bacterial suspensions were standardized with McFarland’s 0.5 Barium Sulfate Standard Solution (approximately 1.5 × 10^8^ CFU mL^−1^) and diluted to 10^6^ CFU mL^−1^. Growth kinetics were evaluated at 19 °C. Standardized bacterial suspensions (500 μL) were inoculated into a flask containing 500 mL of fresh FMM broth. The flasks were placed on a shaker with constant agitation at (110 rpm), and absorbance readings were taken hourly at 620 nm. Simultaneously, samples were seeded onto Petri dishes containing FMM agar, and dilutions were adjusted based on the increase in absorbance to establish the correlation of CFU/mL with optical density.

### 2.4. In Vitro Stimulation of Head Kidney Macrophages (HKMs)

Adherent Atlantic salmon monocytes/macrophages were isolated from the head kidneys of 15 fish smoltified Atlantic salmon obtained from a Puerto Montt hatchery (described in Section 2.2). Of these, five fish were used to standardize the cell isolation protocol, differentiation conditions, and macrophage purity assessment and were not included in gene expression analyses. The remaining ten fish were allocated to two independent experiments: five fish for the dose–response analysis and five fish for the time course analysis. The tissue was mechanically dissociated using a 100 µm cell strainer (Falcon, Corning Inc., Corning, NY, USA) with the aid of a plunger and washed with culture medium. The cell suspension was centrifuged at 1500× *g* for 5 min, the supernatant was discarded, and the pellet was resuspended in a minimal volume of medium. Cell concentration was determined by counting using a hemocytometer under an inverted microscope (Nikon, TMS, Japan). Cells were cultured in Leibovitz’s L-15 medium supplemented with L-glutamine and 10% fetal bovine serum (FBS, Gibco, Thermo Fisher Scientific, Waltham, MA, USA) at 15 °C until fully differentiated.

Fully differentiated macrophages were exposed to *T. dicentrarchi* to evaluate immune-related gene expression by qPCR. Two experimental approaches were performed: (1) a dose–response analysis, in which cells were exposed to *T. dicentrarchi* at different multiplicities of infection (MOI 1, 5, 10, and 50) for 12 h, with unstimulated cells serving as negative controls; and (2) a time course analysis, in which cells were exposed to *T. dicentrarchi* at MOI 5 for 0 (control), 4, 8, 12, and 24 h. In both experiments, head kidney cells were isolated from each fish individually and seeded into five wells of a 6-well plate, with each well receiving a different treatment (MOI or time point). Each fish therefore contributed one independent biological replicate per condition, and individual data points shown in the figures (*n* = 5) represent values from five independent fish.

### 2.5. Gene Expression Analysis by qPCR

Total RNA was extracted using TRIzol (Invitrogen, Thermo Fisher Scientific, Waltham, MA, USA) following the manufacturer’s instructions. The RNA concentration was quantified using a Nanodrop-1000 spectrophotometer (Thermo Fisher Scientific), and RNA integrity was assessed by visualizing 28S and 18S rRNA bands on 1.5% agarose gels stained with SBYR-Safe DNA gel stain (Invitrogen). cDNA synthesis was performed using the RevertAid First Strand cDNA Synthesis kit (Thermo Fisher Scientific) according to the manufacturer’s protocol. Quantitative PCR was conducted using KAPA SYBR FAST qPCR Master Mix (Merck, Darmstadt, Germany) on an AriaMX real-time PCR system (Agilent, Santa Clara, CA, USA). Expression levels of genes related to immune responses were analyzed, including: pro-inflammatory cytokines interleukin-1 beta (*il1β*), tumor necrosis factor-alpha (*tnfα*), interleukin-8 (*il8*), interleukin-12 (*il12*), and interferon gamma (*ifnγ*); anti-inflammatory cytokines interleukin-10 (*il10*) and interleukin-4/13 (*il4/13*); cytokine receptors interleukin-10 receptor (*il10r*) and interferon receptor 1 (*ifnr1*); and transcription factors T-box transcription factor (*tbet*), GATA binding protein 3 (*gata3*), signal transducer and activator of transcription 6 (*stat6*), and suppressor of cytokine signaling 1 (*socs1*); effector molecules inducible nitric oxide synthase (*inos*) and arginase (*arg*) (Table 1). Elongation factor 1 alpha (*ef1α*) and beta-actin (*β-actin*) served as reference genes for normalization (Table 1) [32,33]. Relative gene expression was calculated using the 2^(−ΔΔCt)^ method [34]. All samples were analyzed in technical triplicate.

**Table 1:** Specific primers used for qPCR. F: forward; R: reverse; Tm: melting temperature (°C). Primer amplification efficiency (E). Accession numbers were obtained from the National Center for Biotechnology Information (NCBI) for Atlantic salmon (*Salmo salar*).

Gene	Primers Sequence	Tm	E	Accession Number/ Reference
*β-actin* (*beta-actin*)	F: AAGATGAAATCGCCGCAC R: ATGGAGGGGAAGACAGCC	60	1.90	[34]
*ef1α* (*elongation factor 1 alpha*)	F: GCTTACAAAATCGGCGGTAT R: CTTGACGGACACGTTCTTGA	60	2.02	[35]
*tnfα* (*tumor necrosis factor-alpha*)	F: GCAGCCATCCATTTAGAGGGTGAA R: CTAAACGAAGCCTGGCTGTAAACG	60	1.90	DQ787157.1
*il10* (*interleukin-10*)	F: ATGAGGCTAATGACGAGCTGGAGA R: GGTGTAGAATGCCTTCGTCCAACA	60	2.07	EF165029.1
*il8* (*interleukin-8*)	F: TGTCGCTGCATTGAGACGGAAA R: AGCGCTGACATCCAGACAAATC	60	1.83	[35]
*il1β* (*interleukin-1 beta*)	F: GTCACATTGCCAACCTCATCATCG R: GTTGAGCAGGTCCTTGTCCTTGA	60	2.00	[35]
*inos* (*inducible nitric oxide synthase*)	F: CCAGCATAAGTGGTTCCAAGACCT R: CCAATCTCAGTGCCCATGTACCAG	60	2.10	XM_035787944.2
*il12* (*interleukin-12*)	F: TTGATAAGGGGGACAGTTTGGT R: TTGTCTAAATCCTCAGCCTCGT	60	1.98	XM_014205516.3
*il4/13* (*interleukin-4/13*)	F: TTGGCCTCCGTGAAAAATGC R: AAGTCTCCTAGCTCCACCT	60	2.10	NM_001204895.1
*il10r* (*interleukin-10 receptor*)	F: CGACTTTCCCTCACTCATTC R: CAGCCACTTGTCTGATATACTT	60	2.05	NM_001141766.1
*arg* (*arginase*)	F: TCACTTTCCACCACCTCTTG R: TCTCCACCGCCTCACGACTC	60	1.99	XM_014190234.3
*gata 3* (*GATA binding protein 3*)	F: CGACGATGTGGATGTACTGTTTA R: TACTATGTGGAGGAGGTGGATAC	60	1.95	NM_001171800.1
*infy* (*interferon gamma*)	F: GTGAGCGGAGGGTGTGGATG R: CAGGAAGTAGTGTTTCTGGGTC	60	1.96	NM_001123558.1
*tbet* (*T-box transcription factor*)	F: GAAGTGAAGGAGGATGGTTCTG R: GTGATGTCTGCGTTCTGATAGG	60	2.06	GU979861.1
*ifnr* (*interferon receptor 1*)	F: TTGGACTGAAGGACCAGGAG R: TTGTCCCCAGGACTCATCTC	60	1.90	NM_001360942.1
*socs1* (*suppressor of cytokine signaling 1*)	F: GTCTTTATAGGCCCACATACC R: CACGACAGCGACAGAATAA	60	2.08	KF699315.1
*stat6* (*signal transducer and activator of transcription 6*)	F: GTTCACGGTAGTCAGGATAAC R: GACCTCCGACATGAACTTAC	60	2.00	XM_014135764.2
*Tenacibaculum dicentrarchi* *16S*	F: ATACTGACGCTGAGGGAC R: TGTCCGAAGAAAACTCTATCTCT	60	2.04	[36]

### 2.6. Immunofluorescence Analysis in SHK-1 Cells

The SHK-1 cell line (adherent macrophages derived from Atlantic salmon head kidney; European Collection of Authenticated Cell Cultures, ECACC 97111106), used at passage 30, was used for immunofluorescence analysis. Cells were cultured in Leibovitz’s L-15 medium at 16 °C and exposed to *T. dicentrarchi* at MOI 5 for 12 h. Unstimulated cells were used as a negative control. Following stimulation, adherent cells were washed three times with 1 X phosphate-buffered saline (PBS) and fixed with 4% paraformaldehyde (PFA) for 10 min at room temperature (RT). Cells were then permeabilized and blocked with a solution containing 3% bovine serum albumin (BSA) and 0.3% Triton X-100 in 1X Tris-buffered saline (TBS) for 30 min at RT. To reduce autofluorescence, cells were treated with quenching solution (50 mM ammonium chloride) for 10 min. Subsequently, samples were incubated overnight at 4 °C with primary monoclonal antibodies against IL-10 and TNFα (diluted 1:100) [37,38]. After three washes with 1X PBS, cells were incubated with Alexa Fluor 488-conjugated secondary antibody (diluted 1:750; Invitrogen) for 90 min in the dark at room temperature. Cell nuclei were counterstained with DAPI. Following final washes with 1X PBS, fluorescence images were captured using a Leica CTR5000 fluorescence microscope (Leica Microsystems, Wetzlar, Germany).

### 2.7. Immersion Challenge with T. dicentrarchi

Prior to the main immersion challenge, a preliminary in vivo dose–response trial was conducted using 45 fish to determine the optimal bacterial concentration required to induce clinical signs of tenacibaculosis. Based on the results of this trial, a concentration of 1.5 × 10^7^ CFU/mL was selected for the main immersion challenge described below.

**Figure 1:**
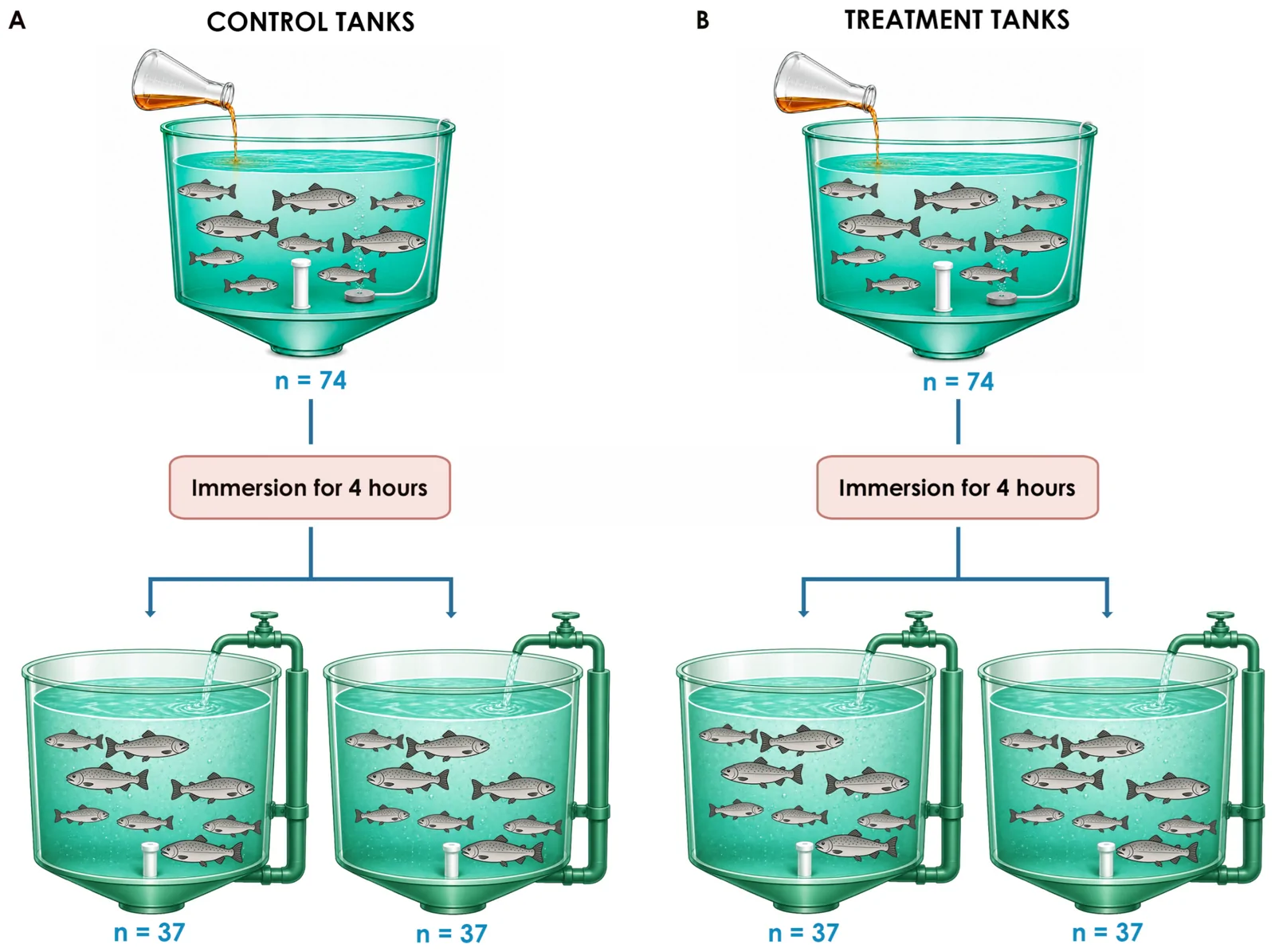
Experimental design of the immersion challenge with *T. dicentrarchi*. (**A**) Control group: sterile FMM medium was added to the tank water, and Atlantic salmon (*n* = 74) were bath-exposed for 4 h, then redistributed into two replicate 500 L recirculating tanks (*n* = 37 fish per tank). (**B**) Treatment group: a *T. dicentrarchi* suspension in FMM medium (1.5 × 10^7^ CFU/mL) was added to the tank water, and Atlantic salmon (*n* = 74) were bath-exposed for 4 h, then redistributed into two replicate 500 L recirculating tanks (*n* = 37 fish per tank). Each treatment was maintained in its own dedicated set of tanks. Fish were monitored daily for 28 days to assess cumulative mortality and the temporal progression of clinical signs, allowing characterization of both the acute and recovery phases of *T. dicentrarchi*-induced disease.

Seventy-four fish per treatment were challenged by immersion bath for 4 h in a single 250 L tank containing either a *T. dicentrarchi* suspension in FMM culture medium (treatment group) or sterile culture medium (control group), at a fish density of 54 kg/m^3^ (Figure 1). Exposure was performed without water flow, with constant oxygen supplementation (100–110% saturation); CO_2_ levels were monitored throughout the trial, at 32 ppt salinity. The bacterial concentration used was 1.5 × 10^7^ CFU/mL, as established from the preliminary dose–response trial described above.

Following the 4 h challenge, fish from each treatment were redistributed into two replicate 500 L recirculating tanks (37 fish per tank; 74 fish per treatment total), at an initial bodyweight of 182 ± 28.4 g and a density of 13.5 kg/m^3^, under normal operating conditions with open water flow. Fish were monitored daily for 28 days, and mortalities were recorded.

To monitor disease progression and confirm bacterial infection, clinical and microbiological assessments were performed throughout the 28-day challenge period. Skin swabs were collected from the lateral body surface of each fish, mouth swabs from the buccal commissures, and gill swabs from the gill filaments, using sterile swabs streaked directly onto FMM plates, then incubated at 17 °C for 72 h. Bacterial presence was further confirmed by Gram staining and qPCR with specific primers for *T. dicentrarchi* (Section 2.5, Table 1). Macroscopic lesions were evaluated in 6 fish per treatment at each sampling point (0, 2, 4, 7, 14, 21, and 28 days post-exposure). At each time point, fish were randomly selected from each tank regardless of health status, euthanized, and permanently removed from the experiment for clinical and bacteriological evaluation. Because their removal was non-informative, these fish were treated as right-censored observations in the survival analysis. Skin tissues were also collected from these fish for bacterial presence. The presence or absence of the following clinical signs was recorded: skin ulcers, mouth damage, frayed fins, gill hemorrhage, systemic hemorrhage, and ocular hemorrhage (defined as visible bleeding in the eye, resulting from escape of blood through vessel walls or by diapedesis [39]).

### 2.8. Statistical Analysis

All statistical analyses and graphical representations were performed using R (version 4.5.1; R Core Team, 2025) within the RStudio integrated development environment (version 4.1.2; Posit Software, PBC, Boston, MA, USA). Prior to analysis, data were evaluated for normality and homogeneity of variances (Shapiro–Wilk and Levene’s tests, respectively). As data did not meet these assumptions, gene expression data were analyzed using the Kruskal–Wallis test and Dunn’s post hoc test to assess significant differences in log fold-change values between groups. Survival curves were compared using the log-rank test (Mantel–Cox). Differences in clinical sign frequencies between infected and control groups were evaluated using Fisher’s exact test. In all analyses, differences were considered statistically significant at *p* < 0.05.

## 3. Results

### 3.1. T. dicentrarchi Triggers a Pro-Inflammatory Response in Atlantic Salmon Head Kidney Macrophages

**Figure 2:**
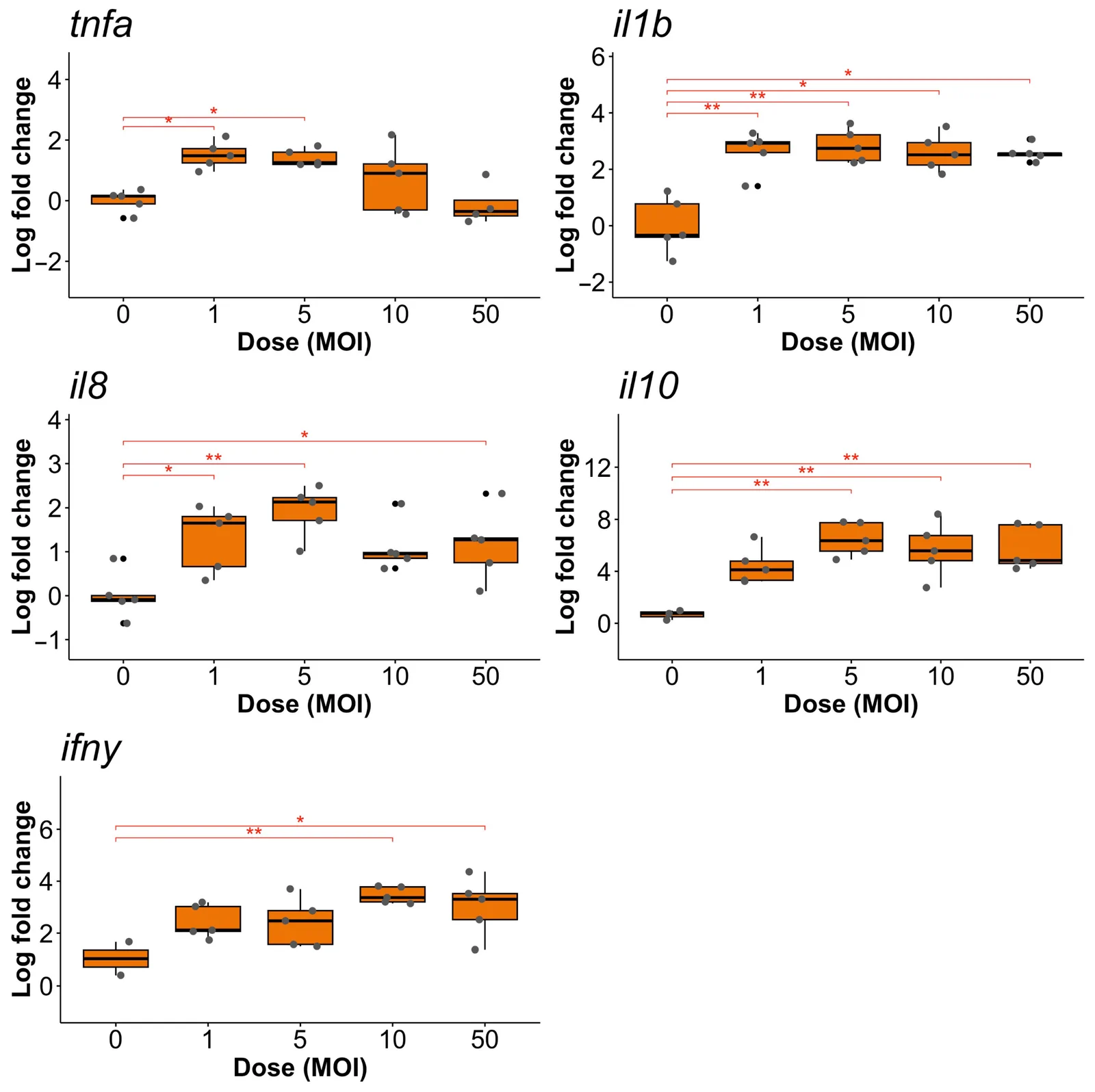
Dose-dependent modulation of immune gene expression in HKM cells exposed to *T. dicentrarchi*. HKM cells were exposed to different multiplicities of infection (MOI 0, 1, 5, 10, and 50) for 12 h. Gene expression was analyzed by qPCR. Data are presented as log fold change relative to unstimulated control (MOI 0). Box plots represent median, interquartile range, and individual data points (*n* = 5). Statistical significance was determined using the Kruskal–Wallis test and Dunn’s post hoc test (* *p* < 0.05; ** *p* < 0.01).

The effects of *T. dicentrarchi* multiplicity of infection (MOI) on immune response markers were evaluated in HKM cells using qPCR. Cells were exposed to *T. dicentrarchi* at MOI 0 (control), 1, 5, 10, and 50 for 12 h. Pro-inflammatory cytokines were significantly upregulated across the different MOIs. *tnfα* gene expression showed significant upregulation at MOI 1 and MOI 5 (*p* < 0.05; Figure 2). Similarly, *il1β* gene expression was significantly elevated at all tested MOIs relative to the control. This increase reached *p* < 0.01 at MOI 1 and 5, and *p* < 0.05 at MOI 10 and 50. *il8*, encoding a key neutrophil chemoattractant, displayed significant upregulation at MOI 1, 5 and 50. The anti-inflammatory cytokine *il10* showed robust induction following bacterial stimulation, with significant increases observed at MOI 5, 10 and 50 (*p* < 0.01). Log fold-change values ranged from 3- to 8-fold, with MOI 5 eliciting the most pronounced response, suggesting an active immunoregulatory mechanism to counterbalance pro-inflammatory signals (Figure 2). *ifnγ* mRNA expression followed a similar trend, with significant upregulation detected at MOI 10 (*p* < 0.01) and MOI 50 (*p* < 0.05) relative to the control.

**Figure 3:**
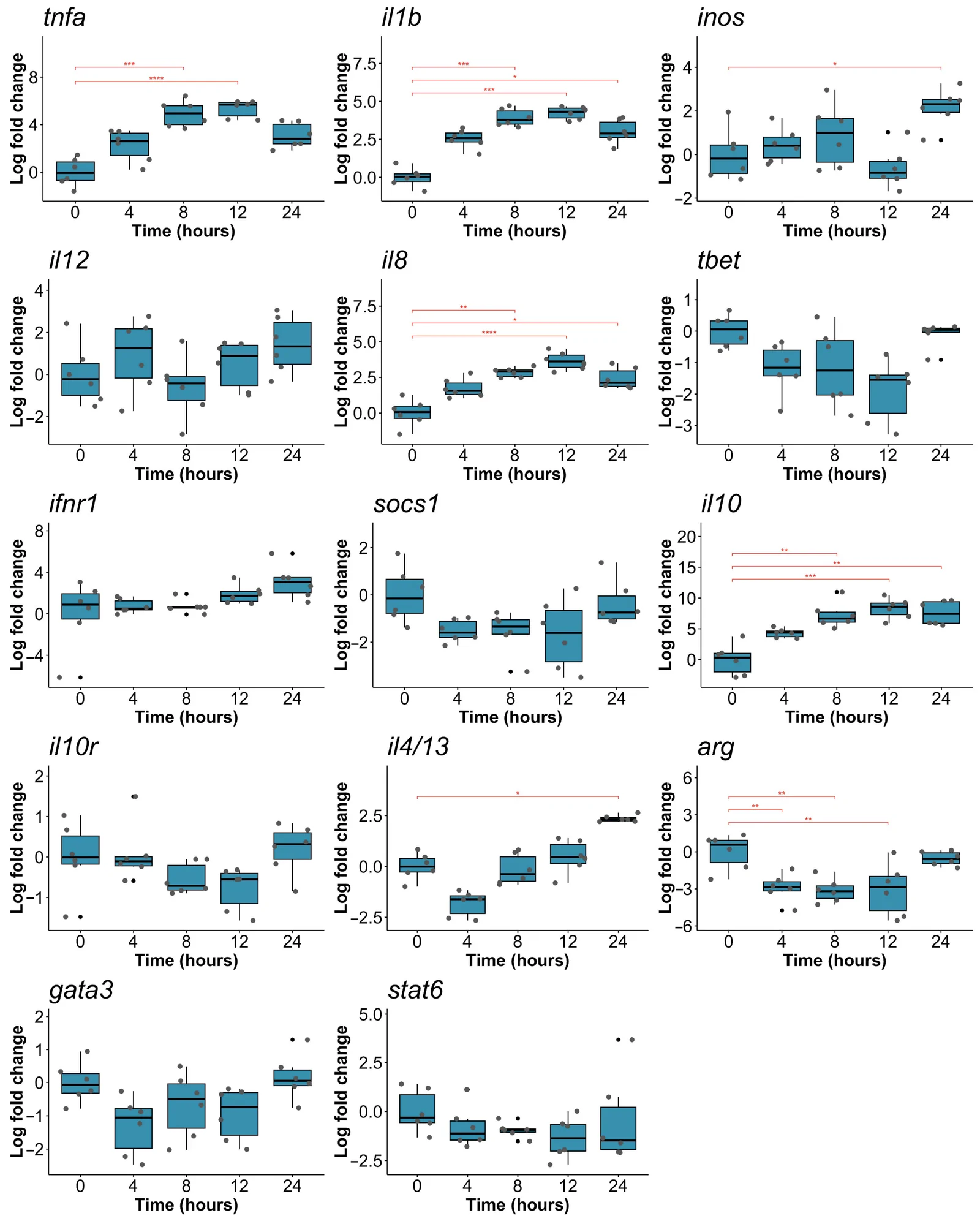
Temporal modulation of immune gene expression in HKM cells exposed to *T. dicentrarchi*. Cells were exposed to *T. dicentrarchi* at MOI 5 and sampled at 0 (control), 4, 8, 12, and 24 h post-exposure (hpe). Gene expression was analyzed by qPCR. Data are presented as log fold change relative to time 0 (unstimulated control). Box plots represent median, interquartile range, and individual data points (*n* = 5). Statistical significance was determined using the Kruskal–Wallis test and Dunn’s post hoc test (* *p* < 0.05; ** *p* < 0.01; *** *p* < 0.001; **** *p* < 0.0001).

Based on these results, MOI 5 was selected to evaluate the temporal dynamics of the immune response in HKM cells exposed to *T. dicentrarchi*. Cells were incubated with the bacterium, and immune marker gene expression was analyzed at 0, 4, 8, 12, and 24 h post-exposure (hpe) by qPCR. The temporal analysis revealed distinct expression patterns among Th1/M1-associated immune markers. The pro-inflammatory cytokine *tnfα* mRNA expression showed a rapid and sustained upregulation following bacterial challenge, with significant elevation observed at 8 and 12 hpe. Similarly, *il1β* and *il8* were both significantly upregulated across the time course, evident at 8 hpe and peaking at 12 hpe, with a slight reduction at 24 hpe (Figure 3). *inos* showed no significant changes in gene expression during the first 12 hpe, followed by significant upregulation at 24 hpe (*p* < 0.05), suggesting a late-phase antimicrobial response (Figure 3).

In contrast, *il12* expression showed limited modulation over the exposure time course, with only marginal changes at intermediate time points (Figure 3). *tbet* expression remained stable across all time points examined, with a slight tendency to be downregulated during the first 12 hpe, followed by recovery of expression at 24 hpe. However, no significant differences were observed compared to the control (Figure 3). *ifnr1* and *socs1*, two genes involved in cytokine signaling regulation, displayed distinct, non-significant kinetic trends across the time course. *ifnr1* expression remained close to baseline from 0 to 8 hpe, followed by a gradual, non-significant upward trend from 12 hpe onward, reaching its highest values at 24 hpe. In contrast, *socs1*, a negative feedback regulator of cytokine signaling, showed sustained downregulation from 4 to 12 hpe, followed by partial recovery toward baseline levels at 24 hpe. The transient suppression of *socs1* during peak cytokine expression (8–12 hpe) could potentially facilitate sustained inflammatory signaling by temporarily reducing negative feedback on cytokine receptor pathways (Figure 3). Overall, *il12*, *tbet*, *ifnr1*, and *socs1* displayed modest and non-significant changes following *T. dicentrarchi* exposure, suggesting limited transcriptional activation of these regulatory pathways under the tested conditions.

The anti-inflammatory cytokine il10 exhibited robust and sustained upregulation throughout the exposure time course. mRNA expression was significantly elevated at 8 hpe (*p* < 0.01), with progressive increases reaching peak levels at 12 hpe (*p* < 0.001) and remaining significantly elevated at 24 hpe (*p* < 0.01) relative to the control. il10r showed a consistent tendency toward downregulation at 4, 8 and 12 hpe; however, no significant differences were detected compared to the control (Figure 3). This tendency toward il10 induction, coupled with a non-significant downregulation trend in il10r, may indicate an immunoregulatory dynamic to counterbalance the strong pro-inflammatory response observed for tnfa and il1β.

In contrast, *il4/13*, which mediates alternative macrophage activation, showed an initial suppression at 4 and 8 hpe, followed by a progressive increase in mRNA expression at 12 and 24 hpe. Statistically significant upregulation was observed only at 24 hpe (*p* < 0.05). *arg* (*arginase*), a hallmark M2 marker involved in polyamine synthesis and tissue repair, showed significant suppression from 4 hpe onward (*p* < 0.01), reaching maximum downregulation at 8 hpe (*p* < 0.01), with suppression remaining significant at 12 hpe (*p* < 0.01), followed by recovery to baseline levels at 24 hpe (Figure 3). This early and sustained downregulation of *arg* is consistent with the relative predominance of M1-like inflammatory activity during the acute phase of exposure. This pattern aligns with the concurrent upregulation of pro-inflammatory markers such as *tnfα* and *il1β*.

*stat6*, the primary transcription factor mediating il4/13 signaling and M2 polarization, exhibited progressive downregulation following *T. dicentrarchi* exposure. Gene expression declined from 4 hpe onward, with sustained suppression observed at 8, 12, and 24 hpe, although these changes did not achieve statistical significance. This non-significant trend toward *stat6* suppression could be consistent with reduced *il4/13* signaling activity, though no firm conclusions can be drawn in the absence of statistical significance. Similarly, *gata3*, the master transcription factor of Th2 responses and a downstream target of *stat6*, showed comparable, non-significant downregulation across most time points. Gene expression was numerically reduced at 4, 8, and 12 hpe, with modest recovery at 24 hpe. The observed non-significant downregulation trend pattern in both *stat6* and *gata3* suggests reduced activity of the alternative macrophage activation pathway, though this requires further experimental validation (Figure 3).

**Figure 4:**
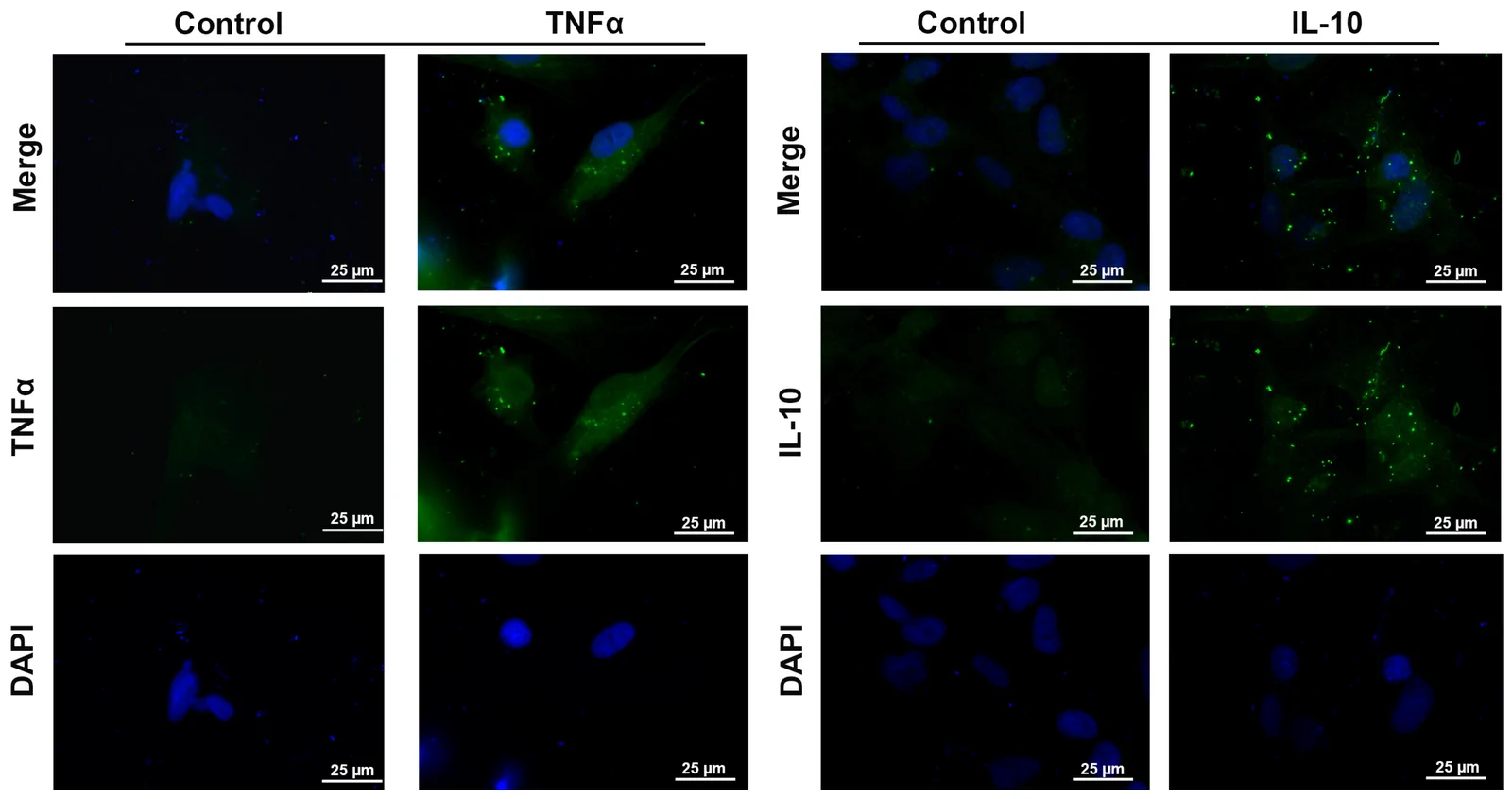
Protein expression analysis of TNF-α and IL-10 in *T. dicentrarchi*-exposed SHK-1 cells by immunofluorescence. Cells were exposed to *T. dicentrarchi* at MOI 5 and analyzed at 12 h post-exposure (hpe). TNF-α expression was detected with an anti-TNF-α antibody, and IL-10 with an anti-IL-10 antibody visualized using a green fluorescent secondary antibody (green). Unstimulated cells were used as a negative control. Nuclei were counterstained with DAPI (blue). Scale bar = [25 µm] applies to all panels. Representative images from *n* = 3 independent experiments.

To confirm the gene expression findings at the protein level, immunofluorescence analysis was performed on SHK-1 cells exposed to *T. dicentrarchi* at MOI 5 for 12 h (Figure 4). TNFα protein was readily detected in exposed cells, showing predominantly cytoplasmic localization with a positive signal, visibly increased compared to unstimulated control cells, suggesting production of this pro-inflammatory mediator (Figure 4). Similarly, IL-10 protein was clearly detected in exposed macrophages, displaying diffuse cytoplasmic distribution (Figure 4), consistent with the transcriptional upregulation observed by qPCR at this time point. Minimal background staining was observed in PBS-treated control cells for both markers, supporting antibody specificity. Collectively, these results confirm protein-level detection of both TNFα and IL-10 in *T. dicentrarchi*-stimulated Atlantic salmon macrophages under the conditions tested, consistent with the coordinated transcriptional response observed in the temporal gene expression analysis.

### 3.2. Immersion Challenge with T. dicentrarchi Induces Mortality and Progressive Clinical Signs in Atlantic Salmon

**Figure 5:**
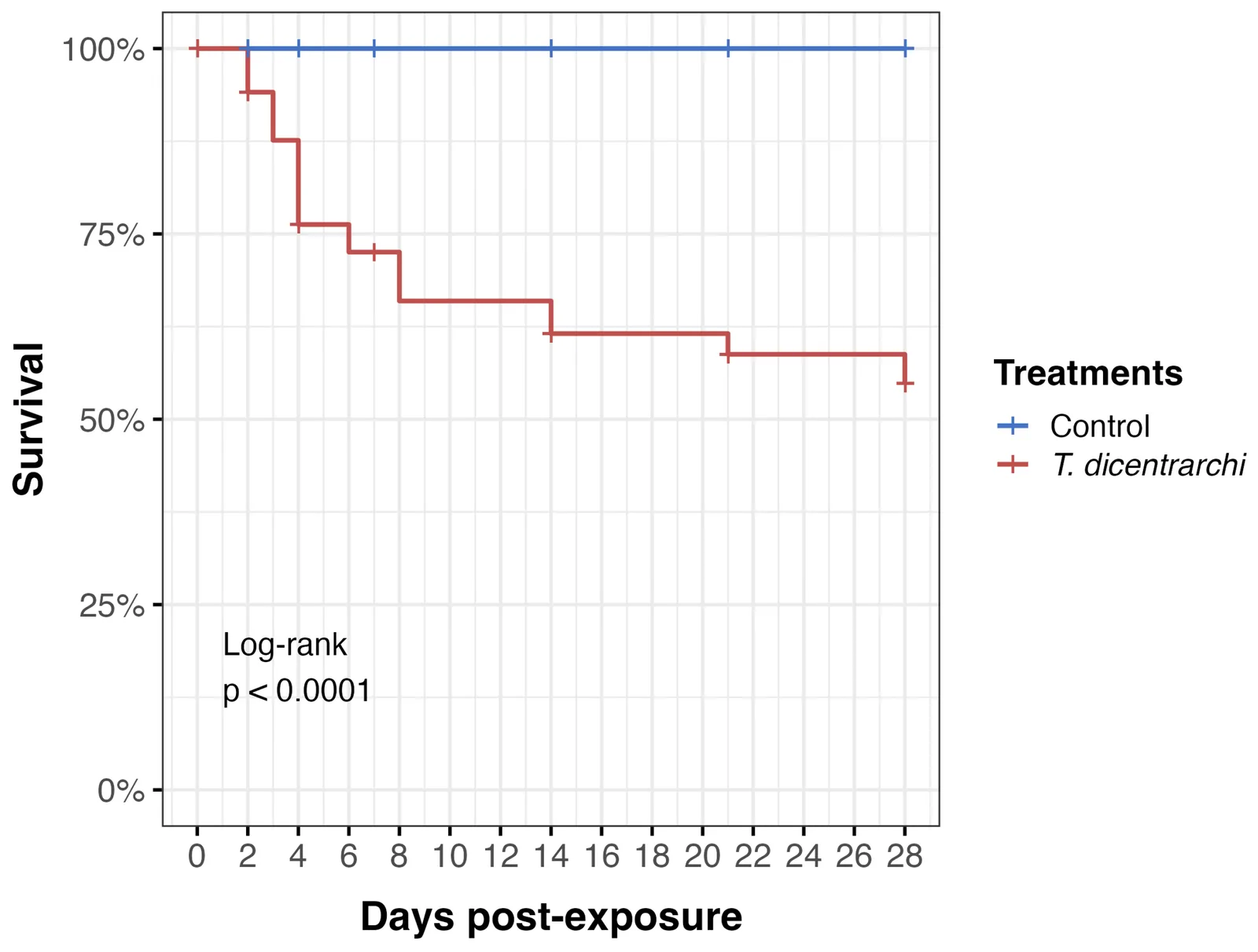
Survival curve of Atlantic salmon challenged with *T. dicentrarchi*. Fish were challenged by immersion with *T. dicentrarchi* at 1.5 × 10^7^ CFU/mL or FMM medium as a control, and monitored daily for 28 days post-exposure (dpe). Survival curves were compared using the log-rank (Mantel–Cox) test. The significant difference between groups: *p* < 0.0001. Data represent *n* = 74 fish per treatment.

To validate the pathogenic potential of a Chilean *T. dicentrarchi* isolate under controlled laboratory conditions, Atlantic salmon were experimentally challenged via immersion exposure. The results demonstrate significant virulence of the bacterial isolate, with challenged fish exhibiting rapid disease progression and mortality (Figure 5). Mortality in the *T. dicentrarchi*-challenged group was first observed at 2 days post-exposure (dpe), with a progressive decline in survival during the initial week. Cumulative mortality reached 30% by day 7, subsequently stabilizing at 32.4% by the end of the 28-day observation period. In contrast, no mortality was observed in the control group (sterile FMM medium) throughout the entire experimental period. Statistical analysis confirmed a highly significant difference between groups (log-rank test, *p* < 0.0001), consistent with high pathogenicity of the *T. dicentrarchi* isolate under experimental immersion conditions (Figure 5).

**Figure 6:**
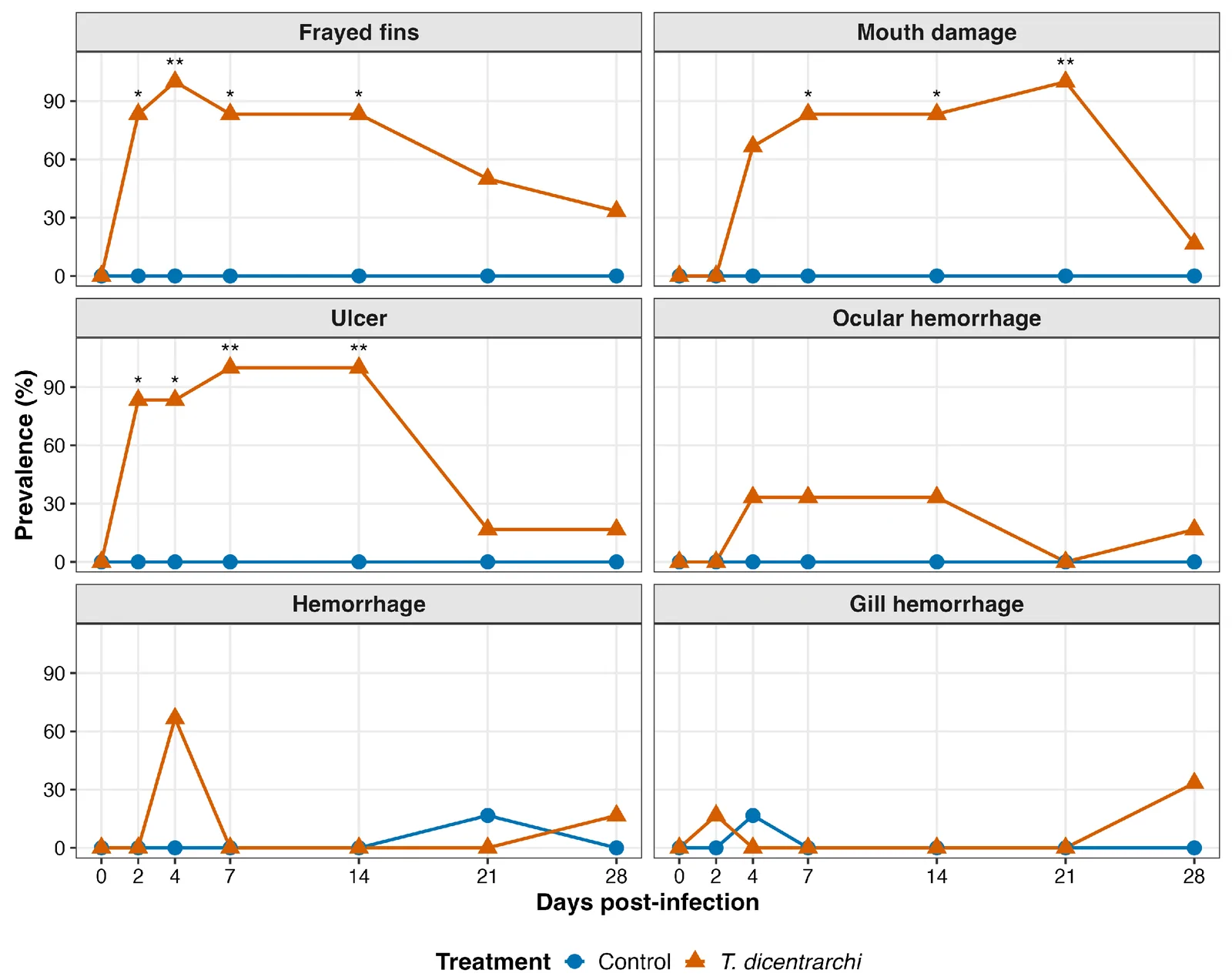
Temporal progression of clinical signs in Atlantic salmon following *T. dicentrarchi* experimental infection. Prevalence (%) of external lesions and tissue damage was monitored at 0, 2, 4, 7, 14, 21, and 28 days post-exposure (dpe) in fish challenged by immersion with *T. dicentrarchi* (orange line) or sterile culture medium (FMM, control; blue line). Clinical signs evaluated included frayed fins, gill hemorrhage, generalized hemorrhage, mouth damage, ocular hemorrhage, and skin ulcers. Data represent the percentage of fish exhibiting each clinical sign at each time point (*n* = 6 fish per treatment per time point). Statistical significance between challenged and control groups was determined using Fisher’s exact test (* *p* < 0.05; ** *p* < 0.01).

**Figure 7:**
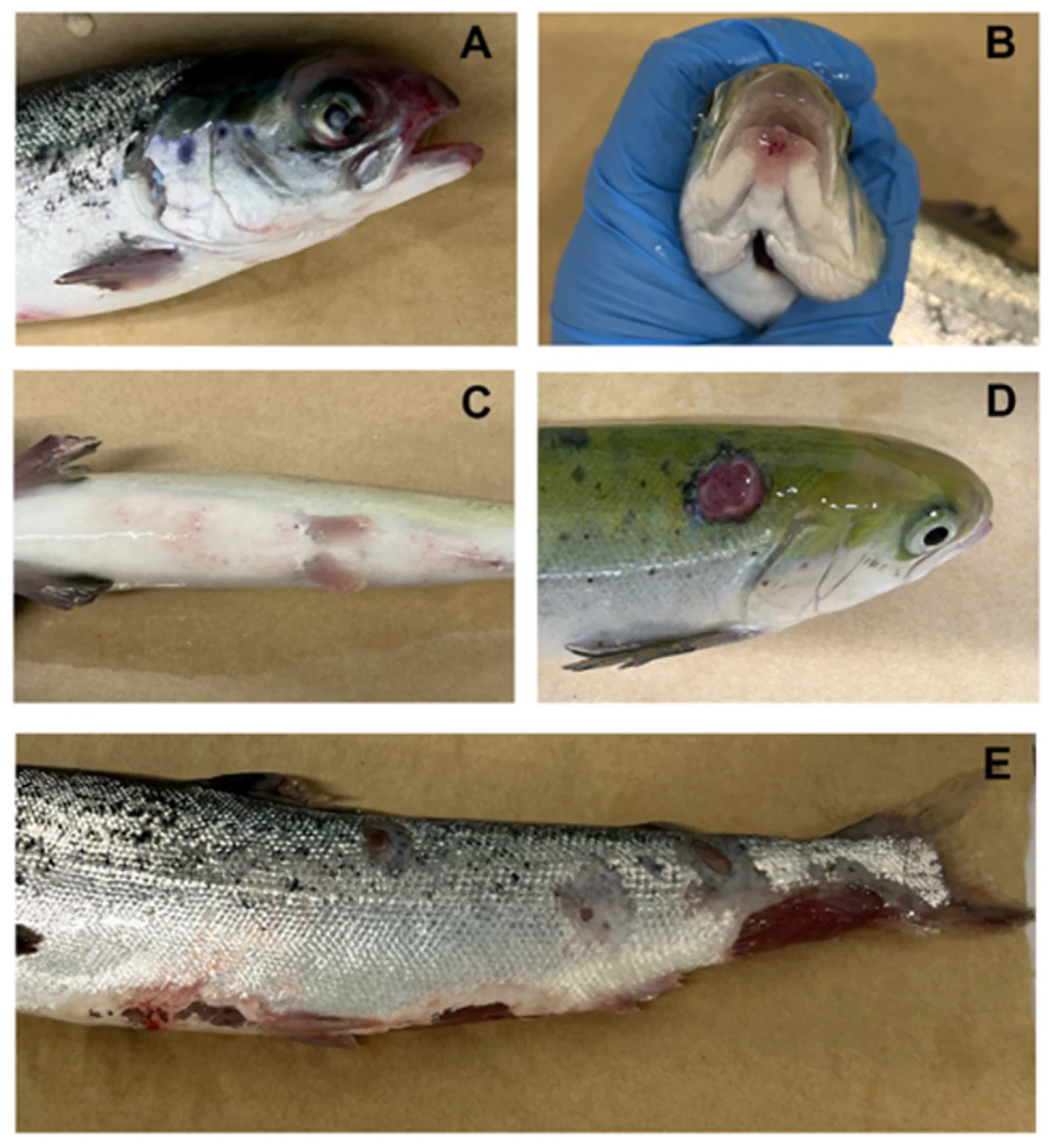
Macroscopic lesions observed in Atlantic salmon challenged with *T. dicentrarchi*. (**A**) and (**B**) mouth damage at 4 dpe; (**C**) skin damage at 4 dpe; (**D**) skin ulcers at 4 dpe; and (**E**) skin ulcers and frayed fins at 14 dpe.

To characterize disease progression and tissue-specific pathology, clinical signs were monitored throughout the 28-day challenge period. Infected fish exhibited a range of external lesions and tissue damage that were absent in control fish, confirming the pathogenic nature of *T. dicentrarchi* infection (Figure 6 and Figure 7). Frayed fins were the earliest and most prevalent clinical sign, appearing as early as day 2 dpe. The prevalence of frayed fins peaked at 4 dpe (100%, *p* < 0.01) and remained significantly elevated through 14 dpe (*p* < 0.05), affecting most challenged fish during this period, with gradual recovery observed at 28 dpe (Figure 6 and Figure 7E). Mouth damage appeared from day 4 dpe (66.7%) and showed a progressive increase, reaching peak prevalence at 21 dpe (100%, *p* < 0.01). A notable decline was observed at 28 dpe (16.7%), suggesting partial recovery at later stages of challenge (Figure 6 and Figure 7B). Skin ulcers emerged rapidly, first detected at 2 dpe (83.3%) and reaching peak prevalence at 7 and 14 dpe (100%, *p* < 0.01). A marked reduction was observed from 21 dpe onward, with prevalence declining to approximately 16.7% by 28 dpe (Figure 6 and Figure 7D,E). Ocular hemorrhage was observed at a consistently low but sustained prevalence of 33.3% between 4 and 14 dpe, with a slight decline thereafter (Figure 6 and Figure 7A). Generalized hemorrhage was observed transiently, with a peak at 4 dpe (66.7%), followed by a rapid decline to negligible levels between 7 and 14 dpe, and a modest increase at 28 dpe (16.7%). Control fish showed occasional hemorrhage only at 28 dpe, though at low prevalence (Figure 6). Gill hemorrhage was detected at low prevalence in challenged fish, with a peak at 2 dpe (16.7%), followed by resolution through most of the experimental period and a modest increase at 28 dpe (33.3%). Gill hemorrhage was observed in one case in control fish at 4 dpe, though without statistical significance (Figure 6). Taken together, these clinical findings are consistent with the ulcerative and hemorrhagic nature of tenacibaculosis in Atlantic salmon. Bacterial etiology was further supported by the recovery of *T. dicentrarchi* from all dead fish, with bacterial presence confirmed by qPCR, Gram staining, and plate bacterial growth on marine medium in both dead and sampled fish.

## 4. Discussion

Tenacibaculosis was officially classified in Chile as a high-risk disease in 2017 and currently ranks as the second leading infectious cause of mortality in Atlantic salmon farming [40]. Among the etiological agents, *T. dicentrarchi* is regarded as the most virulent species and accounts for the majority of clinical cases, while *T. maritimum* and *T. finnmarkense* contribute only marginally to outbreaks [40]. In this study, we examined the response of Atlantic salmon head kidney macrophages; key effector cells of the innate immune system, following in vitro stimulation with *T. dicentrarchi*, and evaluated disease progression using an immersion challenge. Selecting an appropriate stimulation dose and duration for the in vitro assays required considering how *Tenacibaculum*-derived products behave across host cell models, since prior studies have reported a range of immunostimulatory and cytotoxic effects depending on the bacterial component and cell type used. To date, in vitro studies on *Tenacibaculum*–host cell interactions have employed soluble extracellular products (ECPs) from *T. maritimum* on epithelial cells [41], outer membrane vesicles (OMVs) from *T. dicentrarchi* on rainbow trout head kidney macrophages [31], live *T. finnmarkense* on Atlantic salmon scale explants [42], and *T. dicentrarchi* in fish mucus models [20]. Collectively, these approaches have shown that ECPs induce epithelial apoptosis, OMVs cause macrophage cytotoxicity, and live *T. finnmarkense* upregulates inflammatory genes in scales, indicating that *Tenacibaculum*-derived products can trigger both protective immune activation and cytotoxic damage depending on dose and exposure time [31,41,42]. Based on the dose–response results (Section 2.4), we selected MOI 5 at 12 h, since it produced a robust pro-inflammatory gene expression profile among the conditions tested, and used this condition for the subsequent temporal assays. The present study is thus the first to characterize the direct immunomodulatory effects of whole live *T. dicentrarchi* on Atlantic salmon head kidney macrophages.

In our in vitro study, *T. dicentrarchi* triggered an early and sustained upregulation of *tnfα*, *il1β*, and *il8*, a pattern consistent with the classical proinflammatory innate response against extracellular Gram-negative bacteria. Comparable cascades have been described in a spleen stromal-like cell line stimulated with *Aeromonas salmonicida* [43], in salmonid intestinal epithelial cells challenged with *Vibrio anguillarum* [44], and in juveniles infected with *Flavobacterium psychrophilum* [45], with responses emerging between 4 and 24 h post-exposure (hpe). This shared pro-inflammatory pattern across Gram-negative fish pathogens is most likely driven by TLR/NOD1-NFκB signaling, the canonical recognition route for Gram-negative bacterial components such as lipopolysaccharide and peptidoglycan [46,47]. Consistent with our results, in vivo infection of European sea bass with *T. maritimum* elicited a similar inflammatory wave 4 to 48 hpe, with marked upregulation of *tnfα*, *il1β*, and *il8* in head kidney and of *il1β* and *il8* in gills, skin, and intestine [24,25], supporting the conserved nature of this response across *Tenacibaculum* species and host models.

Inducible nitric oxide synthase (iNOS) is a hallmark effector molecule of classically activated (M1) macrophages, mediating bacterial killing through nitric oxide (NO). In our model, *inos* was significantly upregulated at 24 hpe, indicating a delayed antimicrobial effector response that follows the early cytokine wave. A similar late *inos* induction has been reported in the SHK-1 cell line at 4 days post-infection and in gill and head kidney tissues at 24 h post-challenge with intracellular pathogens such as *Piscirickettsia salmonis* and *Renibacterium salmoninarum*, where NO production represents a key mechanism for pathogen clearance [48,49]. LPS derived from Gram-negative bacteria is also a known inducer of *inos* gene expression in salmonids, catfish, and carp [50,51,52], and iNOS-mediated NO production has been reported in mammalian responses to extracellular pathogens such as *Pseudomonas aeruginosa* in mammalian models [53], suggesting a conserved antimicrobial strategy across both intracellular and extracellular bacterial challenges.

In parallel with this proinflammatory cascade, *il10* was robustly and continuously upregulated from 4 to 24 hpe, indicating the simultaneous engagement of immunoregulatory mechanisms aimed to counterbalance inflammation. IL-10 is a key anti-inflammatory cytokine that limits immunopathology during bacterial infections, and a similar temporal profile has been reported in European sea bass mucosal tissues challenged with *T. maritimum*, where *il10* expression was elevated from 6 to 48 h post-challenge [24]. The concurrent induction of *tnfα*, *il1β*, and *il10* observed here therefore reflects a balanced response in which the host attempts to clear the pathogen while limiting collateral tissue damage, a pattern increasingly recognized as characteristic of effective immunity in teleosts [47]. Interestingly, despite robust *il10* upregulation, interleukin-10 receptor (*il10r*) showed a consistent tendency toward downregulation throughout the time course. This trend could suggest a regulatory dynamic in which effector functions are maintained while preventing excessive immunosuppression. Immunofluorescence analysis confirmed the protein-level detection of both TNF-α and IL-10 proteins in *T. dicentrarchi*-exposed Atlantic salmon macrophages, consistent with the dual transcriptional signature observed in the temporal analysis.

In contrast to this coordinated M1-associated and immunoregulatory response, two key marker genes of alternative macrophage (M2) activation were modulated in the opposite direction. *il4/13* was initially downregulated at 4 hpe and progressively recovered toward 24 hpe, while *arg* was significantly downregulated at 4, 8, and 12 hpe. IL-4/13 drives Th2 differentiation and alternative (M2) macrophage activation [54], whereas arginase competes with iNOS for the common substrate L-arginine, redirecting L-arginine metabolism toward polyamine and collagen synthesis associated with tissue repair rather than NO-mediated killing [55]. The simultaneous suppression of *arg* and late induction of *inos* suggests that *T. dicentrarchi* exposure actively shifts L-arginine metabolism toward NO production. This metabolic competition, referred to as the “arginine fork” [56], is a key determinant of M1/M2 balance in teleosts, and its resolution in favor of iNOS in the present model further supports the predominance of an inflammatory profile in this in vitro model.

Collectively, these data suggest that *T. dicentrarchi* exposure induces a dominant M1-like inflammatory profile, accompanied by IL-10-mediated immunoregulation and a relative reduction in M2-associated gene expression, a profile consistent with the extracellular Gram-negative nature of the pathogen and with TLR/NF-κB-driven activation. Importantly, this profile also helps explain the severe tissue damage characteristic of tenacibaculosis: a sustained inflammatory response, even when partially counterbalanced by IL-10, may drive the progressive ulcerative lesions and tissue destruction observed in vivo. Some limitations of the in vitro component must, however, be acknowledged. Primary macrophage monocultures cannot recapitulate the architectural complexity of intact head kidney tissue or the systemic context of infection, including stress hormones, individual genetic variation, and co-infections prevalent under field conditions [57,58,59]. The pathogen–macrophage interaction examined here therefore represents a controlled mechanistic approximation rather than a full reflection of host–pathogen dynamics in the living fish. Despite these constraints, in vitro models remain essential tools in fish immunology, both for dissecting cellular infection mechanisms and for alignment with the 3R principles governing the ethical use of animals in research [60]. 

To evaluate disease outcome under controlled experimental conditions, we developed an immersion challenge model using the same isolate. The trial yielded a cumulative mortality of 32.4%, with first deaths recorded at day 2 and a peak at day 4, consistent with the rapid disease progression previously reported for *T. dicentrarchi* in salmonids in immersion challenges [2,9,61]. Direct comparisons among challenge studies remain challenging due to differences in bacterial concentration, exposure duration, fish size, and isolate virulence. Nevertheless, our mortality rate falls within the reported range: Avendaño-Herrera et al. (2016) reported 65% mortality after a 1 h immersion with *T. dicentrarchi* at 3.78 × 105 CFU/mL; intraperitoneal injection of *T. dicentrarchi* at 1 × 10^6^ cells/mL produced 50% mortality in Atlantic salmon [61], a comparable mortality outcome achieved via a route that bypasses the mucosal and epithelial barriers relevant to natural infection and is therefore not directly comparable in terms of effective bacterial dose; and immersion at 2 × 10^7^ cells/mL caused 72% mortality in Chinook salmon [8], supporting the existence of species-dependent differences in susceptibility among salmonids and/or pathogenicity differences among isolates used.

Tenacibaculosis is primarily an external ulcerative disease affecting the skin, fins, mouth, and gills, with occasional systemic dissemination in severely affected individuals. Consistent with this pattern, challenged fish in the present study exhibited a range of external lesions: frayed fins, skin ulcers, mouth damage, gill hemorrhage, and ocular hemorrhage, confirming the successful reproduction of tenacibaculosis under our experimental conditions. The temporal progression of clinical signs provides insight into the kinetics of the infection. The early appearance of frayed fins at 2 dpe, reaching 100% prevalence by 4 dpe, suggests that fin surfaces represent the primary colonization site, likely facilitated by mechanical damage associated with handling and crowding [2,9]. Skin ulcers, present from day 2 and peaking between days 7 and 14, reflect the progressive destruction of the epithelium and underlying dermis, described histologically as complete loss of epidermis and dermis with superficial muscle necrosis and a mild-to-moderate peripheral inflammatory response [62,63]. The later peak in mouth damage at day 21 is consistent with progression from surface colonization to more protected mucosal surfaces, as previously described for Chilean isolates [2]. Compared with Kumanan et al. [8], who reported musculature exposure in 51% of Chinook salmon challenged with *T. dicentrarchi* at a similar bacterial concentration, the lower severity of deep tissue damage observed here may reflect species- or isolate-dependent virulence differences, highlighting the importance of host–pathogen specificity in determining disease outcome.

A particularly notable finding was a decline in lesions by day 28, with skin ulcer prevalence declining from 100% at day 14 to approximately 16.7% at the end of the observation period, a population-level decline in lesion prevalence among sampled surviving fish that has rarely been documented because most challenge studies are terminated between 8 and 14 days post-exposure [8,23]. The lower prevalence observed at day 28 may reflect adaptive immune activation in surviving fish, combined with implicit selection of more resistant individuals, as cumulative mortality was concentrated in the first week of the challenge. The transient generalized hemorrhage, peaking at day 4 and resolving rapidly thereafter, establishes a direct mechanistic link between the in vitro and in vivo findings of the present study. The peak pro-inflammatory cytokine response, particularly the sustained upregulation of *tnfα* and *il1β* at 8–12 hpe in head kidney macrophages, likely underlies the systemic vascular damage manifested as generalized hemorrhage in the whole animal, while the concurrent and robust *il10* induction may contribute to its subsequent resolution by dampening excessive inflammatory signaling [41,64]. Finally, the sustained ocular hemorrhage observed between 4 and 14 dpe represents an infrequently reported clinical sign in salmonid tenacibaculosis and warrants further histopathological investigation, as it may reflect either a specific characteristic of the Chilean isolate employed or heightened vascular susceptibility of Atlantic salmon to this pathogen compared to other salmonid species previously studied.

Taken together, the immersion challenge developed here, which produced 32.4% cumulative mortality and a clinical profile consistent with field tenacibaculosis, provides a useful experimental platform for studying disease progression over several days and for future evaluation of vaccine candidates and therapeutic strategies against *T. dicentrarchi* in Chilean Atlantic salmon aquaculture.

## 5. Conclusions

This study provides the first integrated in vitro and in vivo characterization of the Atlantic salmon response to *T. dicentrarchi*. Stimulation of head kidney macrophages with live bacteria at MOI 5 elicited an M1-like inflammatory response, with early and sustained upregulation of *tnfα*, *il1β*, and *il8*, late induction of *inos*, and concurrent downregulation of M2-associated genes (*il4/13*, *arg*). A robust *il10* response, together with the protein-level detection of both TNFα and IL-10 in macrophage cultures upon *T. dicentrarchi* exposure, revealed a balanced pro- and anti-inflammatory program. The immersion challenge reproduced the clinical features of field tenacibaculosis, with 32.4% cumulative mortality and partial lesion resolution by day 28. This model provides a controlled platform for evaluating host–pathogen interactions and for future testing of vaccines or therapeutic interventions against *T. dicentrarchi*.

## Data Availability

The data will be made available on request.

## Abbreviations

The following abbreviations are used in this manuscript:

ANID	Agencia Nacional de Investigación y Desarrollo
*arg*	*arginase*
BSA	bovine serum albumin
CFU/mL	colony-forming units per milliliter
DAPI	4′,6-diamidino-2-phenylindole
dpe	days post-exposure
ECPs	extracellular products
*ef1α*	*elongation factor 1 alpha*
FBS	fetal bovine serum
FMM	Flexibacter maritimus medium
*gata3*	*GATA binding protein 3*
HKM	head kidney macrophage
hpe	hours post-exposure
*ifnγ*	*interferon gamma*
*ifnr1*	*interferon receptor 1*
*il1β*	*interleukin-1 beta*
*il4/13*	*interleukin-4/13*
*il8*	*interleukin-8*
*il10*	*interleukin-10*
*il10r*	*interleukin-10 receptor*
*il12*	*interleukin-12*
IL-10	interleukin-10 (protein)
iNOS	inducible nitric oxide synthase
IPNV	Infectious Pancreatic Necrosis Virus
ISAV	Infectious Salmon Anemia Virus
M1	classically activated macrophage polarization
M2	alternatively activated macrophage polarization
MOI	multiplicity of infection
NCBI	National Center for Biotechnology Information
NF-κB	nuclear factor kappa B
NO	nitric oxide
OMVs	outer membrane vesicles
PBS	phosphate-buffered saline
PCR	polymerase chain reaction
PFA	paraformaldehyde
qPCR	quantitative polymerase chain reaction
RAS	recirculating aquaculture systems
RT	room temperature
*socs1*	*suppressor of cytokine signaling 1*
*stat6*	*signal transducer and activator of transcription 6*
TBS	Tris-buffered saline
*tbet*	*T-box transcription factor*
TLR	Toll-like receptor
TNF-α	tumor necrosis factor-alpha (protein)
